# WTAP-Mediated N6-Methyladenosine Modification Promotes Gastric Cancer Progression by Regulating MAP2K6 Expression

**DOI:** 10.7150/jca.98559

**Published:** 2025-01-27

**Authors:** Shuangshuang Han, Haibin Jiang, Jia Wang, Chao Li, Ting Liu, Mingda Xuan, Bo Tian, Yi Si, Hongyan Zhao, Yunxia Zhao, Zhenlong Zhu, Weifang Yu, Lihong Wang

**Affiliations:** 1 Department of Endoscopy Center, The First Hospital of Hebei Medical University, Shijiazhuang, China.; 2 Department of Gastroenterology, Chengde Central Hospital, Chengde, China.; 3 Department of Internal Medicine, The First Hospital of Hebei Medical University, Shijiazhuang, China.; 4 Department of Pathology, The First Hospital of Hebei Medical University, Shijiazhuang, China.

**Keywords:** WTAP, N6-Methyladenosine (m^6^A), MAP2K6, Gastric Cancer, Cell Biology

## Abstract

Wilms tumor 1 associated protein (WTAP) is a key RNA N6-methyladenosine (m^6^A) methylase, which is involved in gastric cancer (GC) development, but its pathogenic mechanism is not clear. This study aims to thoroughly explore the underlying molecular mechanism of WTAP-mediated m^6^A modification in GC pathogenesis. qRT-PCR and immunohistochemistry showed that significantly elevated WTAP expression in GC tissues and is related to advanced age, poorly differentiation, lymph node metastasis and high TNM stage. Overexpression and knockdown of WTAP could promote or inhibit the proliferation, migration and invasion of GC cells *in vitro*, furthermore, suppression of WTAP expression impeded the growth of xenograft tumors *in vivo.* Utilizing RNA sequencing, methylated RNA immunoprecipitation (MeRIP) sequencing and bioinformatics analysis, we identified MAP2K6 as direct downstream target of WTAP with m^6^A modification in GC. The interaction between WTAP and MAP2K6 was confirmed by MeRIP-qPCR, luciferase reporter assay, Co-IF and bioinformatics prediction. Immunofluorescence and rescue studies were performed to verify WTAP-mediated m^6^A modification promotes the proliferation, migration, and invasion of GC cells by positively regulating the target gene MAP2K6. This underscores the potential therapeutic significance of targeting the WTAP-MAP2K6 axis in combating GC occurrence and progression.

## Introduction

Gastric cancer (GC) is one of the most common malignant tumors worldwide. According to relevant research statistics, GC ranks the fifth and fourth among malignant tumors in terms of morbidity and mortality worldwide 1. In China, it ranks both third in the morbidity and mortality of malignant tumors 2. This is undoubtedly a tremendous threat to the health of all mankind. However, it is a pity that the mechanism of occurrence and development of GC is not fully clarified so far. This may be attributed to its intricate mechanisms, mainly involving the interaction between oncogenes and tumor suppressor genes 3, 4, genetics, epigenetics and environmental factors, etc 5, 6. Further exploring the mechanism of occurrence and progression of GC will be of utmost help to the early detection of GC, improve the prognosis of GC patients, and reduce the burden of family and society.

In recent years, more and more researchers have paid attention to the role of N6-methyladenosine (m^6^A) modification in tumor pathogenesis. m^6^A modification is the most common RNA epigenetic modification on mRNA in eukaryotes, especially in higher eukaryotes. It is enriched in the 3' -untranslated region (UTR) near the mRNA stop codon and has a common sequence of RRACH (R=G or A; H=A, C or U) 7, 8. m^6^A modification can regulate RNA stability, translation efficiency, alternative splicing and localization at the post-transcriptional level 9, 10. m^6^A related proteins can be divided into three categories: methyltransferase, demethylase and reader protein 11, 12. Wilms tumor 1 associated protein (WTAP), a methylated transferase, is an essential regulator of the m^6^A methyltransferase complex, which interacts with METTL3 and METTL14 to assist its localization in the nucleus 13, 14. Previous studies have shown that WTAP can promote the occurrence and development of many types of cancer, such as the expression levels of WTAP in esophageal cancer, hepatocellular carcinoma and colon adenocarcinoma were significantly increased 15-17; WTAP promoted the proliferation of renal cell carcinoma by regulating the stability of CDK2 mRNA 18; WTAP played a role in promoting diffuse large B-cell lymphoma by combining with Hsp90 and BCL6 forming a complex 19. Nevertheless, there are few studies on the correlation between WTAP and GC at present.

It was observed that WTAP expression in GC tissues was visibly more increased than that in adjacent normal tissues not only in TCGA and GEO databases but also in cancer and adjacent tissues of 50 pairs of GC patients. Furthermore, our research group also clarified that the ability of GC cells to proliferate, migrate, and invade were enhanced or weakened after WTAP was overexpressed or knocked down respectively. At the same time, we observed that the growth of GC xenografts *in vivo* was significantly inhibited when WTAP expression level was knocked down in GC cells. Subsequently, we employed high-throughput sequencing in conjunction with bioinformatics analysis to identify MAP2K6 as the downstream target gene regulated by WTAP in an m^6^A-dependent manner in GC. We then utilized experimental techniques such as MeRIP-qPCR, luciferase reporter assay, and Co-IF to validate the interaction between WTAP and MAP2K6. More significantly, knocking down or overexpressing MAP2K6 could respectively counteract the promotional or inhibitory effects of WTAP overexpression or knockdown on cell proliferation, migration, and invasion. In conclusion, our research suggests that targeting the WTAP-MAP2K6 axis may represent a novel therapeutic strategy for the treatment of GC.

## Materials and Methods

### Patients and samples

A total of 50 pairs of GC tissues and adjacent normal tissues were taken from GC patients. Inclusion Criteria: No history of other malignant tumors; no preoperative adjuvant treatments such as radiotherapy or chemotherapy; complete clinical case data. Exclusion Criteria: History of other malignant tumors; preoperative tumor resection at another facility or receipt of other adjuvant treatments; incomplete clinical case data; other reasons deemed unsuitable for inclusion by the researchers. These tissues were all pathologically confirmed as GC and normal gastric mucosal tissue postoperatively, and all patients signed an informed consent form. These samples were fixed with 4% paraformaldehyde (Shijiazhuang Huawo Kerui Biological Technology Co., Ltd., Hebei, China) or placed in liquid nitrogen for short-term storage within 5 min after isolation.

The research was approved by the Ethics Committee of the First Hospital of Hebei Medical University (Approval Letter No.: 20200344).

### Cell culture

Human gastric mucosa cell (GES-1) and GC cells (HGC-27, AGS, MKN7, MKN74) were obtained from Wuhan Pricella Biotechnology Co., Ltd., and were verified by STR analysis. Both cell lines were regularly tested formycoplasma contamination. All cells were cultured in RPMI 1640 medium (Gibco, Gaithersburg, MD, USA) supplemented with 10% fetal bovine serum (FBS; Gibco) and 1% penicillin-streptomycin-gentamicin solution (Solarbio Sciences & Technology Co., Ltd., Beijing, China). Incubation was carried out in a humidified incubator at 37°C with 5% carbon dioxide.

### Plasmids, siRNAs, shRNAs, transfections and stable cell lines

Cells were digested in 25 cm^2^ cell culture flasks after they reached 80-90% fusion, then the WTAP overexpression plasmid, WTAP siRNA (GenePharma Co., Ltd., Shanghai, China), WTAP shRNA (GeneCopoeia Co., Ltd., Guangzhou, China), Mitogen-activated protein kinase kinase 6 (MAP2K6) siRNA (GenePharma Co., Ltd.), MAP2K6 overexpression plasmid and their respective negative control were transfected into GC cells for 6 h by using Lipofectamine 2000 (Invitrogen, Carlsbad, CA, USA) according to its instruction. After the cells were harvested, the transfection efficiency was detected by qRT-PCR and Western blot. For the construction of stable transfected cell lines (HGC-27 Sh-ctrl, HGC-27 Sh-WTAP), neomycin with a final concentration of 600 μg/mL was added into the culture medium without antibiotics 48 hours after transfection, and the concentration of neomycin was adjusted to 300μg/mL after clones were formed.

### Cell proliferation assay

Cells of each group were seeded uniformly in 96-well plates at a density of 1×10^3^/well, and cultured in cell incubator with complete culture medium. Then, 10μL Cell Counting Kit-8 (CCK-8; Dojindo, Tokyo, Japan) reagent was added to each well at 24 h, 48 h, 72 h and 96 h after seeding. The absorbance of each well at 450 nm was measured using a Promega GloMax luminescence detector (Promega, Madison, WI, USA) after the cells added with CCK-8 reagent were incubated in the cell incubator for 2 h.

### Colony formation assay

GC cells of each group were seeded (1000 cells per well) in a 6-well plate and then cultured for consecutive 10 days. Then, after washing with PBS, 4% paraformaldehyde was applied to the colonies, and 0.1% crystal violet was applied to them. Distinct colonies with at least 50 cells were visually counted.

### Wound healing assay

Cells from each group were seeded evenly in 6-well plates at a density of 4×10^5^/ well and cultured in cell incubator with complete culture medium. When the cell growth density reached or approached 100%, a straight line was scratched in each hole with the tip of the pipette to simulate a wound. Each well was photographed at 0 h, 24 h and 48 h after scratch formation, and the cell migration distance was expressed as the ratio of the gap width measured at 48 h to that measured at 0 h.

### Transwell migration and invasion assay

Migration and invasion assays were conducted using Transwell chambers (Corning Incorporated, Corning, NY, USA). For the migration assay, 3×10^4^ cells were suspended in 200 μL RPMI 1640 medium without FBS and added to the upper chamber. The lower chamber was filled with 700 μL complete culture medium to induce chemotaxis. In the invasion assay, the upper chamber contained 100 μL medium, while the lower chamber contained 500 μL medium; all other conditions remained unchanged from the migration assay. After 24 hours of incubation, cells on the upper chamber side of the membrane were swabbed and stained using the Diff-Quick staining kit (Solarbio Sciences & Technology Co., Ltd.). Five random fields were selected for photography, and stained cells were quantified using Image J software (National Institutes of Health, Bethesda, MD, USA).

### RNA sequencing and methylated RNA immunoprecipitation (MeRIP) sequencing assay

Extracted total RNA from WTAP knockdown HGC-27 cells, and entrusted BGI Genomics Co.,Ltd. (Shenzhen, China) for library construction and sequencing. This experiment was divided into two groups: Sh-ctrl and Sh-WTAP. For RNA sequencing, a total of six samples were tested by using DNBSEQ platform, the average output of each sample was 6.70 G. For MeRIP sequencing, different libraries were sequenced on Illumina Nova platform. MeRIP-Seq enriched the region of m^6^A modification on RNA and sequenced it. Therefore, the number of reads covered by IP library in the region of m^6^A modification was significantly higher than that of INPUT library, thus forming a "peak". The location of these peaks indicates which regions of RNA are m^6^A modified. After the m^6^A modification was identified, annotation, distribution statistics and motif identification were performed for it.

### Animal studies

Ten 5-week-old male BALB/c nude mice, procured from Beijing Huafukang Biotechnology Co., LTD (Beijing, China), were randomly allocated into two groups and housed in a pathogen-free environment with ad libitum access to water and food. Subsequently, 5 × 10^6^ HGC-27 cells engineered with stable transfection of shRNA-NC and shRNA-WTAP, were subcutaneously injected into the left hind limb flank of the nude mice. Tumor size was assessed every 2 days starting from the 4th day post-injection. Mice were humanely euthanized when tumor volume approached 1000 mm^3^. Tumor specimens were harvested, weighed, fixed in formalin, and subsequently subjected to paraffin embedding and sectioning for HE and IHC analysis. The above experiments were approved by the Ethics Committee of the First Hospital of Hebei Medical University.

### RNA extraction and qRT-PCR

Total RNA was extracted from gastric cell lines and frozen tissues according to the instruction of RNA-easy Isolation Reagent (Vazyme Biotech Co.,Ltd., Nanjing, China). Then RNA was reversely transcribed into cDNA using PrimeScript RT reagent kit (Takara, Beijing, China). qRT-PCR was then performed using AceQ Universal SYBR qPCR Master Mix (Vazyme Biotech Co.,Ltd.), β-actin was used as endogenous control of mRNA. The primer sequences were displayed in supplementary Table 1. Three sub-wells were set for each group of samples. The expression levels of mRNA were defined based on CT (cycle threshold) values for each sample, and the relative expression levels of samples were calculated by 2-∆∆CT method.

### Immunohistochemistry (IHC)

Tissues from patients and nude mice were fixed with 4% paraformaldehyde, embedded in paraffin (Shanghai YiYang Instrument Co., Ltd., Shanghai, China), sectioned and stained. IHC analysis was performed using the Rabbit two-step assay kit (Beijing ZSGB-Bio Co., Ltd., Beijing, China) according to the manufacturer's instructions. Anti-WTAP (diluted 1:100; Abcam, Cambridge, MA, USA; Catalog number: ab195380) and Anti-Ki-67 (diluted 1:50; Proteintech, Wuhan, Hubei, P.R.C; Catalog number: 27309-1-AP) was used for IHC staining. The positive results were determined by the staining intensity and the proportion of positive cells. The result determination method is as follows: to observe and detect the degree of diffuse homogeneous light yellow fine particles, yellow particles and brown yellow coarse particles in the corresponding parts of the tissue, 0 points for no coloring, 1 point for weak coloring, 2 points for moderate coloring, and 3 points for strong coloring. In addition, the percentage of positive cells in the visual field is scored: 1 point for ≦25%, 2 points for 25%-50%, 3 points for 51%-75%, and 4 points for 76%-100%. Multiply the two scores as the final judgment result, ≦3 is divided into the negative group, > 3 is divided into the positive group.

### Immunofluorescent assay

GC cells and tissue sections were first incubated with anti-WTAP (diluted 1:200), anti-MAP2K6 (diluted 1:200; Proteintech Group, Inc.), and then they were respectively incubated with secondary antibody (Cy3 anti-rabbit IgG, FITC anti-mouse IgG; Beyotime Biotechnology Co., Shanghai, China). Finally, 4'6'-diamidino-2-phenylindole dihydrochloride (DAPI; Beyotime Biotechnology Co.) was added to stain nuclei. Representative images were taken by a fluorescence microscopy or a laser scanning confocal microscope (Olympus, Tokyo, Japan).

### Western blot analysis

Total protein was isolated from the cultured cells using RIPA lysis buffer (Beyotime Biotech Co., Ltd., Shanghai, China). Proteins were separated by 12% SDS polyacrylamide gel electrophoresis (Shanghai Epizyme Biotechnology Co., Ltd., Shanghai, China) and transferred onto polyvinylidene difluoride membranes (Merck Millipore, Billerica, MA, USA). The immunoreactive protein bands were detected by the Odyssey Scanning System (LI-COR Biosciences, Lincoln, NE, USA) after using antibodies against WTAP (diluted 1:1500; Abcam, Cambridge, MA, USA; Catalog number: ab195380) and β-actin (diluted 1:1500; Solarbio Sciences & Technology Co., Ltd.; Catalog number: K101527P).

### MeRIP-qPCR

Total RNA was extracted from HGC-27 cells transfected with siRNA-NC/siRNA-WTAP and from AGS cells transfected with pcDNA3.1-vector/pcDNA3.1-WTAP, and immunoprecipitation was performed according to the instructions of the MeRIP kit (Sigma-Aldrich, Merck, Germany; Catalog number: 17-10499). Subsequently, the magnetic beads are washed with IP buffer. After RNA elution, the level of target RNA was detected by qPCR for 3 times.

### Luciferase reporter assay

Luciferase reporter plasmids containing wild-type CDS-MAP2K6 or mutant CDS-MAP2K6 were synthesized by Saierbio (Tianjin, China) and cloned into the pmirGLO vector. The HGC-27/AGS cells were co-transfected with the MAP2K6-WT/MAP2K6-Mut luciferase reporter plasmids while knockdown/overexpressing WTAP, and the cells were lysed after 48 hours of culture. Subsequently, the luciferase activity was analyzed and measured using a Dual Luciferase Reporter Assay Kit (RG009, Beyotime) and a GloMax 96 Microplate Luminometer (Promega, USA). The firefly luciferase activity was normalized to the renilla luciferase activity, and the experiment was independently repeated three times.

### Databases analysis

The WTAP gene expression data of 375 GC tissues and 32 adjacent normal tissues were obtained from TCGA on June 5, 2022. And GSE54129 and GSE66229 datasets from the GEO database were used as validation cohorts. The online database NCBI, Database for Annotation, Visualization and Integrated Discovery, Venny 2.1.0, RNA Modification Database V2.0, GeneCards and the software iGV were used to predict potential downstream regulatory target genes of WTAP, PrimerBank and Primer designing tool were used to design primers. The online database TCGA and Gene Expression Profiling Interactive Analysis (GEPIA) were then used to further determine the mRNA expressions of target genes. The online website catRAPID omics (http://s.tartaglialab.com/page/catrapid_group) was used to predict binding site motifs between the WTAP protein and MAP2K6 mRNA.

### Statistical analysis

All the experiments in each group were performed with three replicates. All statistical analyses were processed by GraphPad Prism 8.0 (GraphPad Software, La Jolla, CA, USA) and SPSS 21 (IBM, Armonk, NY, USA). The results of normally distributed data were expressed as mean ± standard deviation and the results of non-normal distribution data were expressed as median and interquartile spacing. In this study, statistical methods such as Student's t-test, one-way ANOVA and two-way ANOVA were applied. When *P*<0.05, the difference was considered statistically significant.

## Results

### The expression level of WTAP in GC tissues was increased

**Figure 1:**
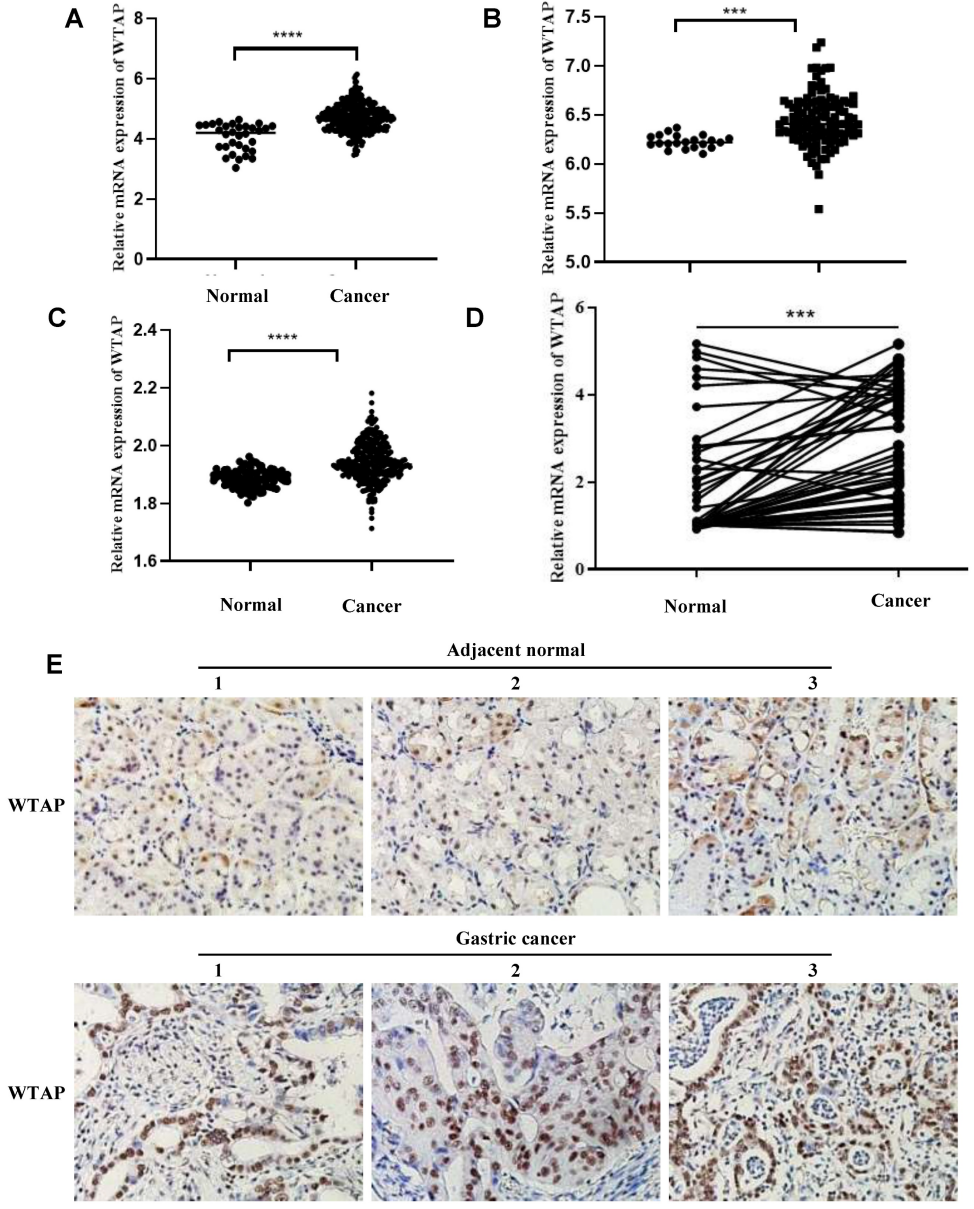
** The expression of WTAP in GC tissues was increased. (A)** WTAP mRNA expression in the pairs of GC and matched adjacent tissues of TCGA Cohort patients; WTAP mRNA expression in the pairs of GC and matched adjacent tissues of GSE54129 **(B)** and GSE66229 **(C)** Cohort patients; **(D)** qRT-PCR analysis of WTAP expression in 50 pairs of GC and adjacent normal tissues. **(E)** Immunohistochemistry was used to detect the expression of WTAP (400X). *** *P* <0.001; **** *P* <0.0001.

To investigate the role of WTAP in GC, we firstly investigated the expression levels of WTAP in GC patients and adjacent normal tissues from TCGA database, GSE54129 and GSE66229 datasets. The results indicated that WTAP was significantly increased in GC tissues of TCGA database, GSE54129 and GSE66229 datasets respectively (Figure 1A-C). In addition, we found that the mRNA level of WTAP was significantly higher in GC fresh tissues than in adjacent normal tissues (Figure 1D), which was also confirmed by the protein level in corresponding paraffin GC tissues and normal tissues by IHC (Figure 1E and Table 1). Combined analysis of WTAP and clinicopathological data displayed that the mRNA expression level of WTAP was associated with advanced age, poorly differentiation and high TNM stage, similarly, the protein expression level of WTAP was related to advanced age, lymph node metastasis and high TNM stage (Tables 2-3). Therefore, the mechanism of WTAP in GC needs to be further explored.

### Overexpression of WTAP promoted the proliferation, migration and invasion of GC cells

**Figure 2:**
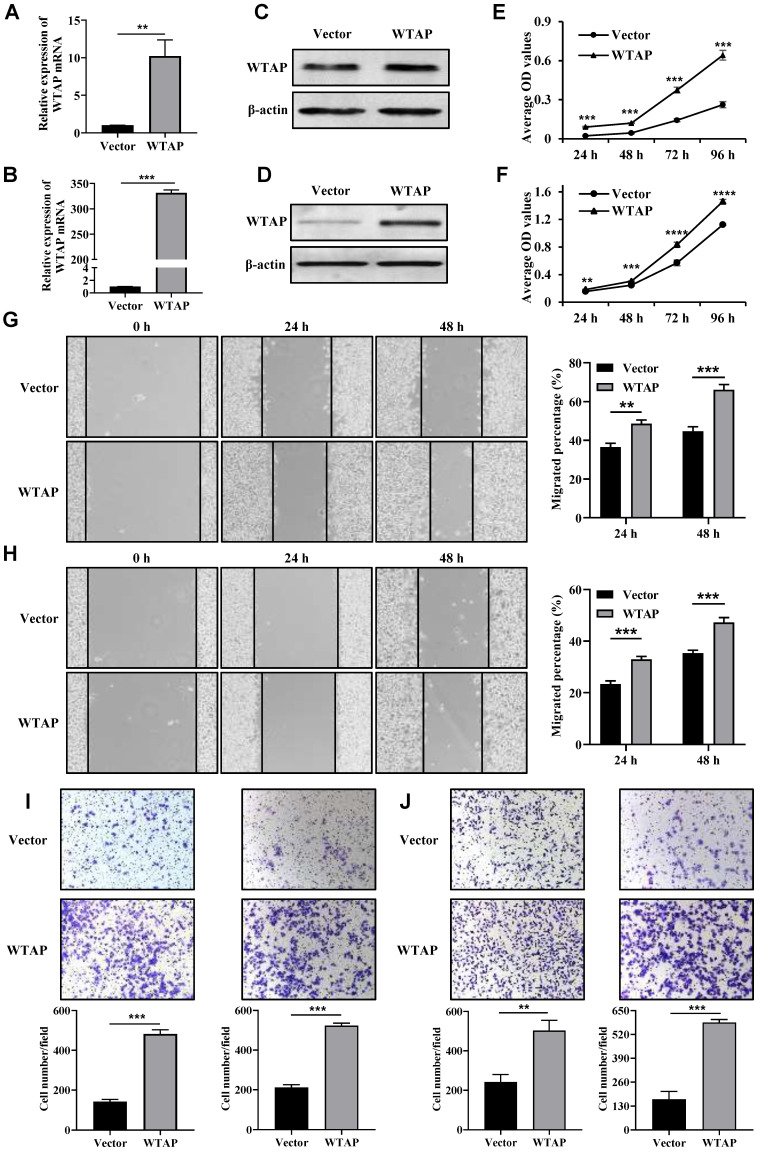
**Overexpressed WTAP promoted the cell proliferation, wound healing, migration and invasion ability.** The overexpression efficiency of WTAP in HGC-27 and AGS cell lines was detected by qRT-PCR **(A, B)** and Western blot analysis **(C, D)**. **(E, F)** The cell viability of HGC-27 and AGS cell lines were determined. **(G, H)** The scratch healing ability of HGC-27 and AGS cell lines was detected. Scale bar, 100 μm. **(I, J)** Transwell migration and invasion assays were used to evaluate HGC-27 and AGS cell migration and invasion ability. Scale bar, 100 μm.** *P* <0.01; *** *P* <0.001; **** P <0.0001.

The expression levels of WTAP protein in wild-type GES-1, HGC-27, AGS, MKN7, and MKN74 cell lines were validated using Western blotting. The results indicate that the expression levels of WTAP protein in the HGC-27 and AGS cell lines are significantly higher than those in human gastric mucosal cells (GES-1) (Supplementary Figure 1). Therefore, HGC-27 and AGS cell lines were chosen as the subjects of the study. The overexpression efficiency of WTAP was confirmed by qRT-PCR (Figure 2A, B) and Western blot analysis (Figure 2C, D). Sequentially, we analyzed the impact of WTAP overexpression on the proliferation of HGC-27 and AGS cells. Compared with the control group, the cell viability was all increased in HGC-27 and AGS cells after transfection with WTAP overexpression plasmid at 24 h, 48 h, 72 h and 96 h (Figure 2E, F). What's more, the migration ability of overexpressed WTAP cells was significantly strengthened compared with the Vector group at 24 h and 48 h (Figure 2G, H). Transwell assays results indicated that migrated and invasive cell numbers in WTAP overexpression group were significantly raised both (Figure 2I, J). These data suggested that overexpression of WTAP significantly promoted cell proliferation, migration and invasion capabilities of GC cells *in vitro*.

### Knockdown of WTAP inhibited the cell proliferation, migration and invasion of GC cells

**Figure 3:**
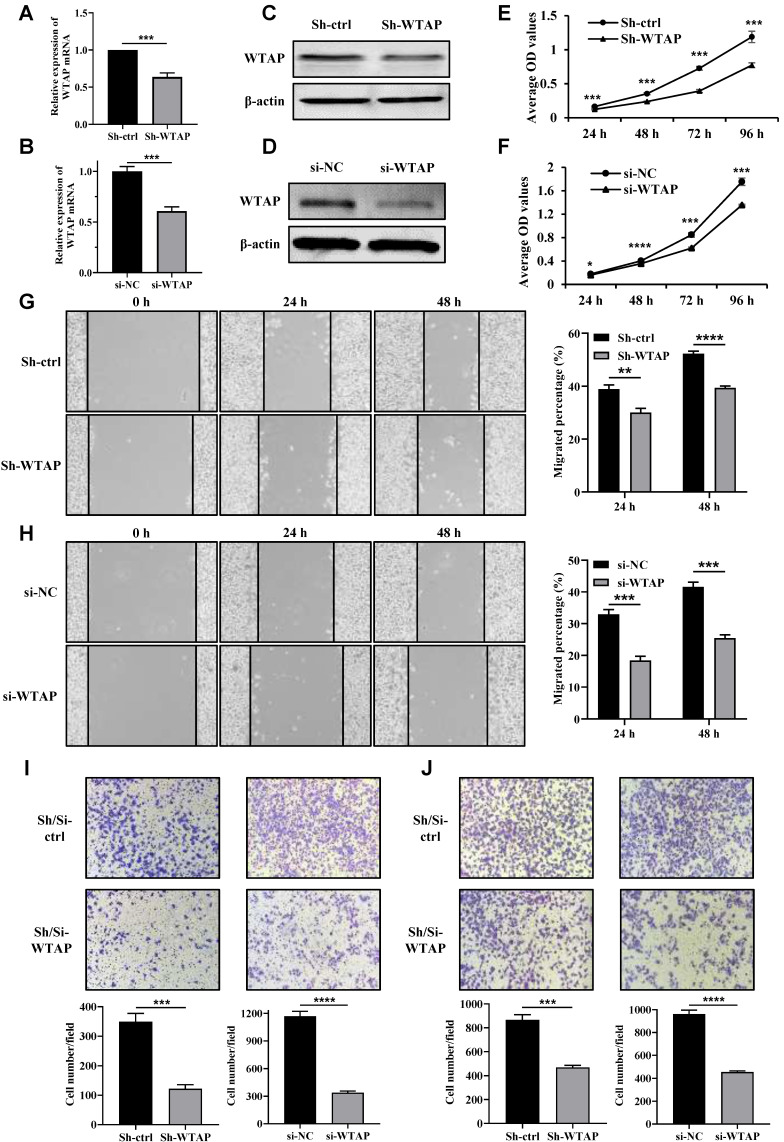
**Knockdown of WTAP inhibited the cell proliferation, wound healing, migration and invasion ability.** The knockdown efficiency of WTAP in HGC-27 and AGS cell lines was detected by qRT-PCR **(A, B)** and Western blot analysis **(C, D)**. **(E, F)** The cell viability of HGC-27 and AGS cell lines were determined. **(G, H)** The scratch healing ability of HGC-27 and AGS cell lines was examined. Scale bar, 100 μm. **(I, J)** Transwell migration and invasion assays were used to evaluate HGC-27 and AGS cell migration and invasion ability. Scale bar, 100 μm.* *P* <0.05; *** *P* <0.001; **** P <0.0001.

Next, we transfected shRNA-WTAP and siRNA-WTAP into HGC-27 and AGS cells separately to knock down WTAP, and then verify the effects of WTAP on the biological functions of the GC cells. The knockdown efficiency of WTAP in GC cells was confirmed by qRT-PCR (Figure 3A, B) and Western blot analysis (Figure 3C, D). The cell viability was obviously down-regulated after WTAP knockdown compared with the control group at 48 h, 72 h and 96 h (Figure 3E, F). Meanwhile, wound-healing assay also suggested that the migration ability of HGC-27 and AGS cells was dramatically attenuated in WTAP-knockdown cells at 24 h and 48 h (Figure 3G, H). Transwell assays results indicated that migrated and invasive cell numbers in the WTAP knockdown group were significantly less than that of the control group in both HGC-27 and AGS cells (Figure 3I, J). Overall, these findings suggested that down-regulation of WTAP significantly suppressed GC cell proliferation, migration and invasion ability *in vitro.*

### Suppression of WTAP expression impeded the growth of xenograft tumors in nude mice

**Figure 4:**
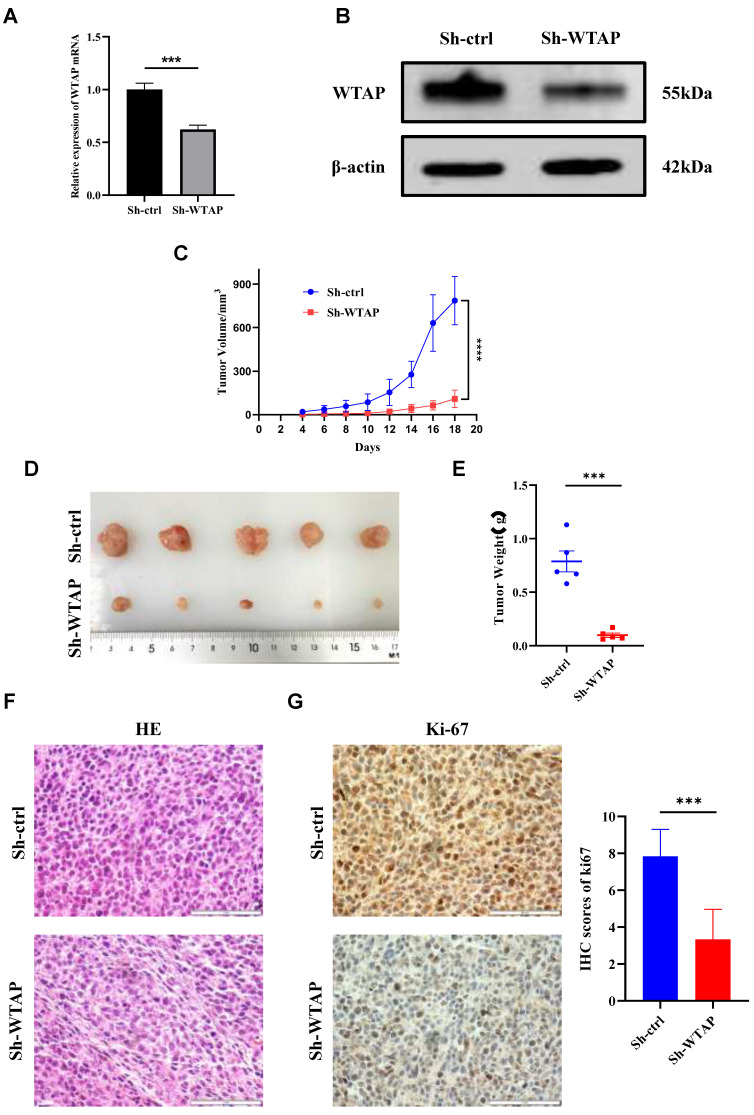
** WTAP regulates the tumorigenesis and growth of GC cells in nude mice. (A)** The expression level of WTAP mRNA in stable cell lines was detected by qRT-PCR. **(B)** The expression level of WTAP protein in stable cell lines was detected by WB. **(C)** The tumor size was measured every 2 days and the tumor growth curve was plotted. **(D)** Tumors of the two groups of nude mice were dissected and photographed on the 18th day after transplantation. **(E)** The tumor was weighed after dissection. **(F)** Representative images of HE staining of the 2 groups of xenograft tumors. Scale bar, 20 μm. **(G)** Representative immunohistochemical results and quantitative analysis of Ki-67 positive staining in 2 groups of xenograft tumors. Scale bar, 20 μm. Data are shown as means ± S.D. *** *P* < 0.001, **** *P* < 0.0001.

To further elucidate the mechanism of WTAP in GC *in vivo*, we established tumor-bearing nude mouse models with low WTAP expression and corresponding negative control. qRT-PCR (Figure 4A) and WB (Figure 4B) results indicated that the mRNA and protein expression levels of WTAP in Sh-WTAP group were lower than those in Sh-ctrl group. The tumor volume curve, tumor photographs, and weights are depicted (Figure 4C-E), revealing significantly smaller tumor volumes and weights in the Sh-WTAP group compared to the Sh-ctrl group. HE staining was utilized to assess xenograft tissue morphological characteristics, demonstrating similarities in cell morphology and structure to GC tissues (Figure 4F). Ki-67 immunohistochemical staining indicated a marked inhibition in proliferation ability of HGC-27 cells with consistently low WTAP expression, in contrast to the control group (Figure 4G). These findings suggest that downregulating WTAP expression can effectively impede GC growth *in vivo*.

### Analysis of transcriptome sequencing and MeRIP sequencing

**Figure 5:**
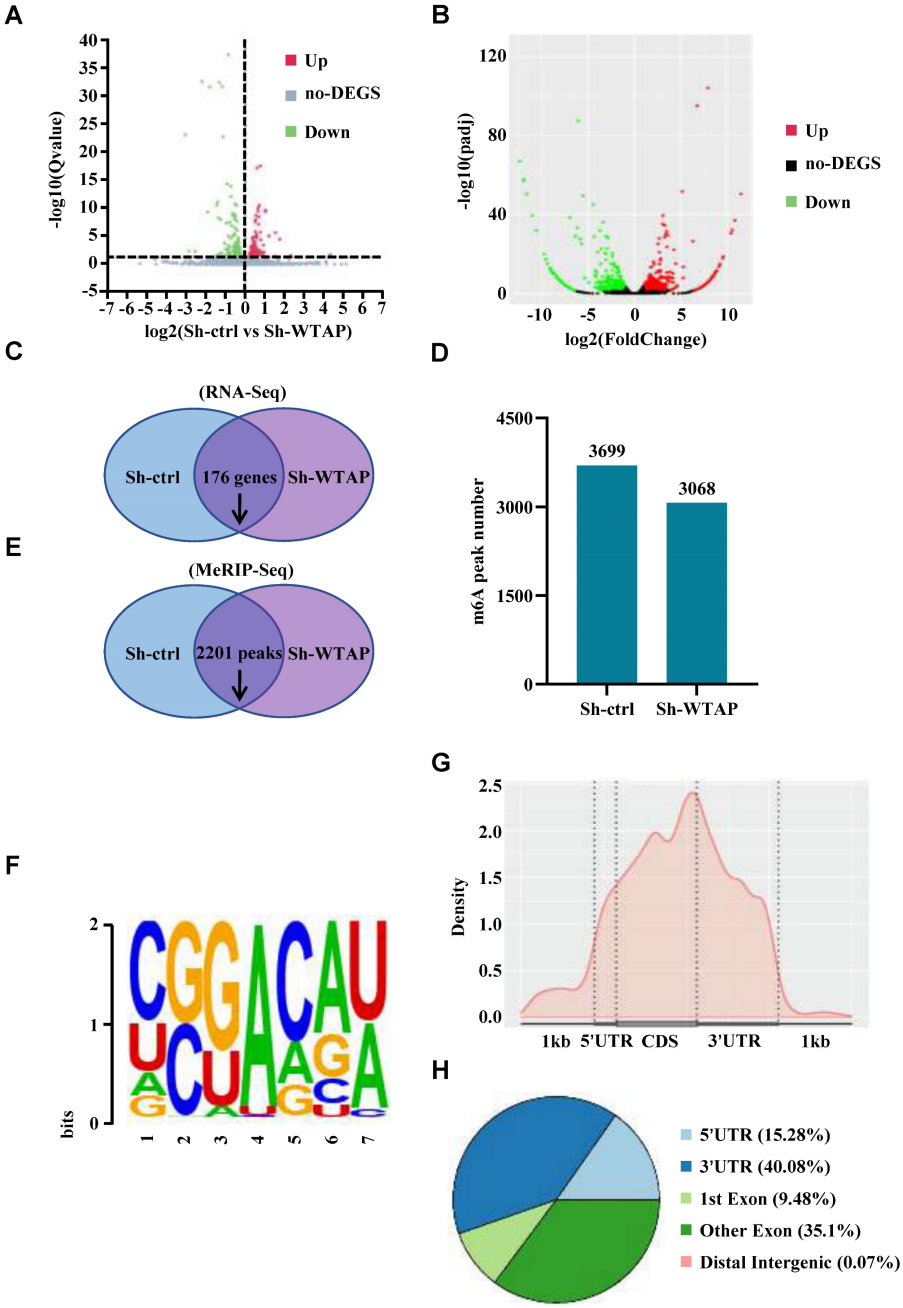
** Analysis of transcriptome sequencing and m^6^A sequencing results. (A-C)** The sequencing results identified the genes significantly changed in Sh-WTAP group compared with Sh-ctrl group. **(D, E)** m^6^A sequencing identified the diminished m^6^A peaks in Sh-WTAP group. **(F)** The m^6^A consensus sequence motif was identified in HGC-27 cells. **(G, H)** Distribution of m^6^A IP signal in mRNA transcripts in HGC-27 cells.

In order to explore the mechanism of WTAP in the pathogenesis and development of GC and further identify the direct downstream target genes of WTAP with m^6^A modification, RNA sequencing and MeRIP sequencing were performed simultaneously in the WTAP-knockdown HGC-27 cell line to find which genes were significantly changed after the downregulation of WTAP. For RNA sequencing, we found that there were 176 genes downregulated and 123 genes upregulated after WTAP knockdown (Figure 5A, C). For MeRIP sequencing, there were 774 downregulated genes and 1003 upregulated genes in the WTAP knockdown group compared with the control group (Figure 5B). Meanwhile, 3699 and 3068 m^6^A peaks were detected in the Sh-ctrl and Sh-WTAP group respectively (Figure 5D). Compared with the control group, 2201 m^6^A peaks were downregulated in Sh-WTAP group (Figure 5E). When mapped the m^6^A methylomes in HGC-27 cells, the m^6^A consensus sequence GGAC (RRACH) motif was identified to be highly enriched within m^6^A sites in the immunopurified RNA (Figure 5F). Moreover, the m^6^A modification was mainly enriched in the CDS and 3'UTR regions of mRNAs (Figure 5G, H). These results indicate that we can carry out the subsequent analysis and validation based on the data obtained by sequencing.

### MAP2K6 was a direct downstream target of WTAP with m^6^A modification

**Figure 6:**
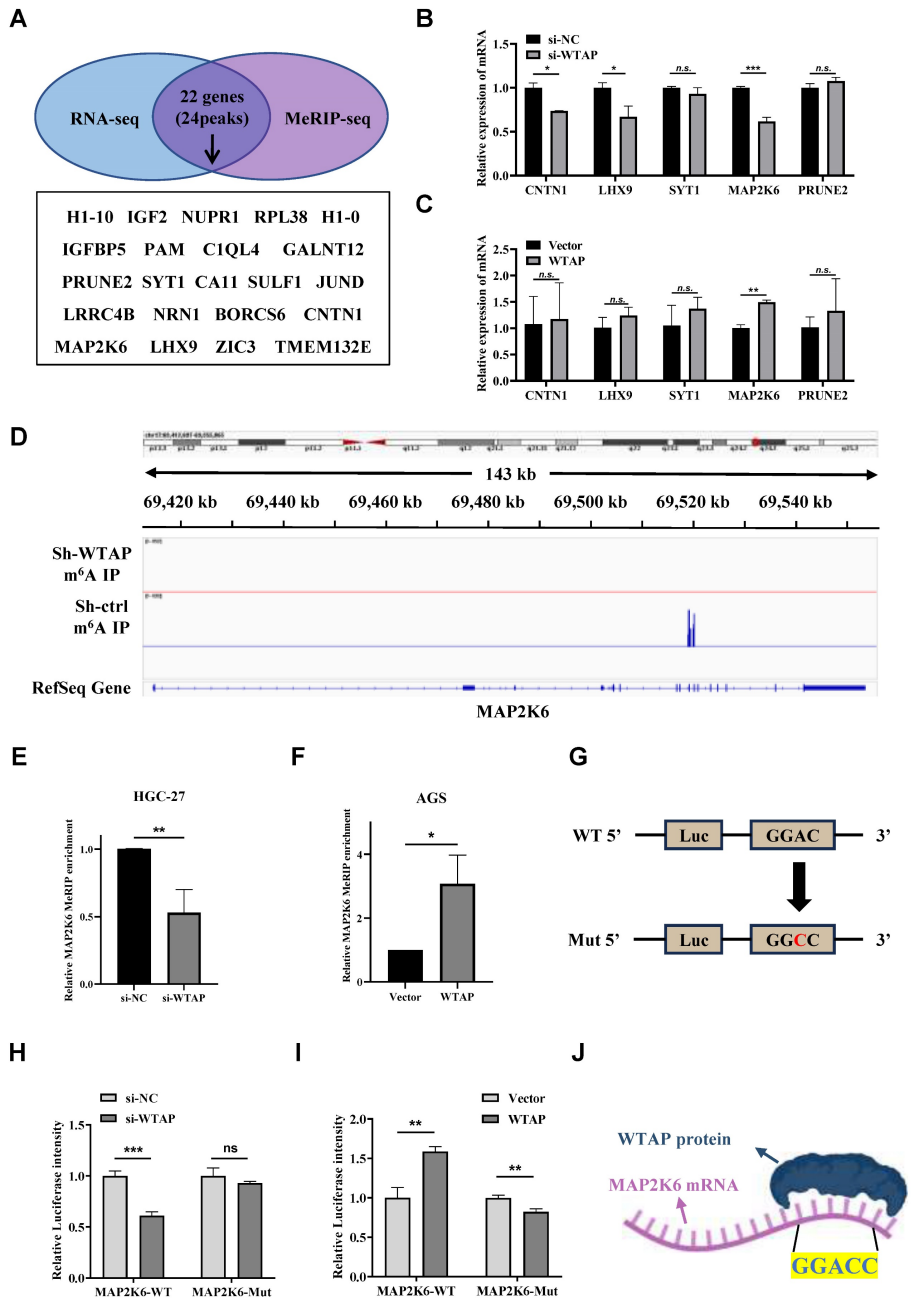
** MAP2K6 is a direct downstream target gene of WTAP. (A)** The intersection of genes corresponding to the downregulated m^6^A peaks in m^6^A sequencing and downregulated genes in transcriptome sequencing. **(B, C)** The expression levels of CNTN1, LHX9, SYT1, MAP2K6 and PRUNE2 genes were detected by qRT-PCR. **(D)** Attenuation of WTAP diminishes m^6^A modification of MAP2K6 mRNA in HGC-27 cells. **(E)** MeRIP-qPCR was used to detect the change of MAP2K6 mRNA enrichment in HGC-27 cells after knocking down WTAP expression level. **(F)** MeRIP-qPCR was used to detect the change of MAP2K6 mRNA enrichment in AGS cells after up-regulating WTAP expression level. **(G)** Diagram illustrating the fusion of the wild-type MAP2K6 sequence and the MAP2K6 sequence with a mutation at the 1409th m^6^A site with the luciferase reporter gene, respectively. (H, I) Relative luciferase intensity of HGC-27/AGS cells under different treatments. **(J)** Schematic diagram of the catRAPID website predicting the binding site between WTAP protein and MAP2K6 mRNA. * *P* < 0.05, ** *P* < 0.01, *** *P* < 0.001.

In order to clarify whether the disappearance or decrease of m^6^A peaks was related to the downregulation of the expression level of some genes, the intersection of 176 downregulated genes obtained by analyzing the above RNA sequencing results and 1818 genes corresponding to 2201 downregulated m^6^A peaks obtained by the above m^6^A sequencing was acquired. 22 genes corresponding to 24 m^6^A peaks were identified (Figure 6A), and the genes with no significant m^6^A peak downregulation and those with m^6^A peak not accurately located in the 3'UTR and CDS regions were eliminated. We then analyzed the subcellular location of genes and discarded genes whose subcellular location was completely unrelated to WTAP. Eventually, we obtained 5 candidate target genes: CNTN1, LHX9, SYT1, MAP2K6 and PRUNE2. After the mRNA expression levels of the 5 candidate target genes mentioned above were analyzed by TCGA and GEPIA2 online websites, a significant difference was found only in MAP2K6 expression between GC tissues and adjacent normal tissues (Supplementary Figure 2). At the same time, we detected 5 candidate target genes in WTAP knockdown HGC-27 cells and WTAP overexpressing AGS cells by qRT-PCR and found that the expression levels of CNTN1, LHX9 and MAP2K6 decreased after knockdown of WTAP, and only the expression level of MAP2K6 was increased with WTAP overexpressing (Figure 6B, C). More importantly, the original two m^6^A peaks of MAP2K6 were both disappeared after the down-regulation of WTAP (Figure 6D). The results of MeRIP-qPCR suggested that when the expression level of WTAP in HGC-27/AGS was down-regulated/up-regulated, the enrichment degree of MAP2K6 mRNA would be correspondingly weakened/enhanced (Figure 6E, F). Then, we constructed luciferase reporter genes containing wild-type and m^6^A site mutant MAP2K6 to further clarify the impact of m^6^A modification on MAP2K6 expression. The m^6^A sites on the MAP2K6 transcript were predicted using the SRAMP database, and the high-scoring site at position 1409 was selected for mutation, changing adenine to cytosine (Figure 6G). Luciferase reporter assays revealed that the transcription level of wild-type MAP2K6 significantly decreased upon WTAP knockdown, while the mutant type did not show a change (Figure 6H). Conversely, WTAP overexpression enhanced the expression of the wild-type MAP2K6 fusion reporter gene, but did not increase the expression of the mutant MAP2K6 (Figure 6I). In conclusion, both MeRIP-qPCR and the dual-luciferase reporter gene assay indicate that the regulation of MAP2K6 expression is controlled by m^6^A modification mediated by WTAP. In addition, the catRAPID website predicted that there was a direct binding site between WTAP protein and MAP2K6 mRNA. The motif of this binding site is GGACC, which is consistent with the previous results of MeRIP-seq (Figure 6J). These findings confirmed that MAP2K6 was a direct downstream target of WTAP with m^6^A modification in GC.

### WTAP positively regulates MAP2K6

**Figure 7:**
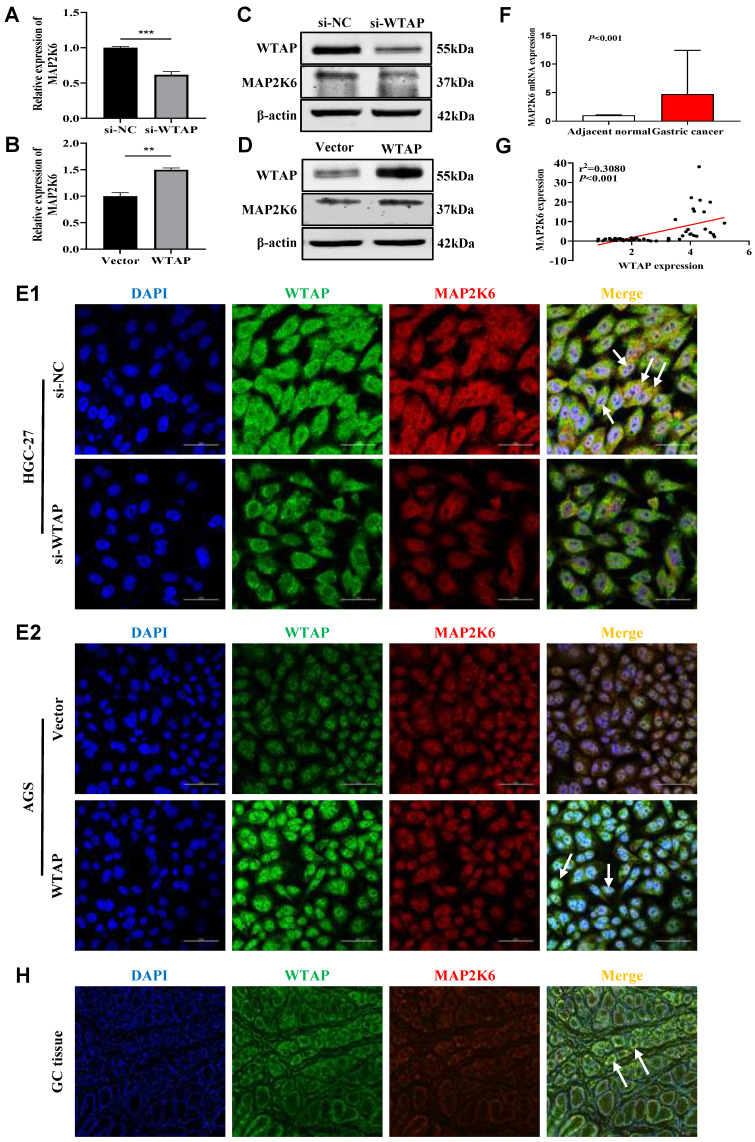
** WTAP positively regulates MAP2K6. (A, B)** The mRNA expression levels of MAP2K6 upon WTAP knockdown or overexpression were measured by qRT-PCR. **(C, D)** The protein expression levels of MAP2K6 upon WTAP knockdown or overexpression were measured by WB. **(E)** Co-expression of WTAP and MAP2K6 in GC cells was determined by confocal immunofluorescent assay (600X). Scale bar, 50 μm. **(F)** The mRNA expression levels of MAP2K6 in 50 GC tissues and paired adjacent normal tissues were determined by qRT-PCR. **(G)** WTAP was positively correlated with MAP2K6 expression in 50 GC tissues. **(H)** The colocalization of WTAP and MAP2K6 in GC tissues were confirmed by immunofluorescent assay (200X). Scale bar, 150 μm.* *P* < 0.05, ** *P* < 0.01, *** *P* < 0.001, **** *P* < 0.0001.

To further confirm the regulatory effect of WTAP on MAP2K6, we knocked down or overexpressed WTAP in GC cell lines, and found that the mRNA and protein expression levels of MAP2K6 were also down-regulated or up-regulated by qRT-PCR (Figure 7A, B) and WB (Figure 7C, D). Immunofluorescence staining showed that the expression of WTAP and MAP2K6 co-localized, and also revealed that when WTAP was knocked down or overexpressed in HGC-27 and AGS cell lines respectively, the expression of MAP2K6 was correspondingly attenuated or enhanced (Figure 7E). Subsequently, qRT-PCR showed that the expression level of MAP2K6 in 50 pairs of human GC tissues was significantly higher than that in adjacent normal tissues (Figure 7F), and there was a positive correlation between its expression level and the expression of WTAP (Figure 7G). Immuno-fluorescence in GC tissues also confirmed the co-localization of WTAP and MAP2K6 (Figure 7H). All the above results indicated that WTAP had a positive regulatory effect on the expression of MAP2K6.

### The oncogenic effect of WTAP is dependent on MAP2K6 in GC cells

**Figure 8:**
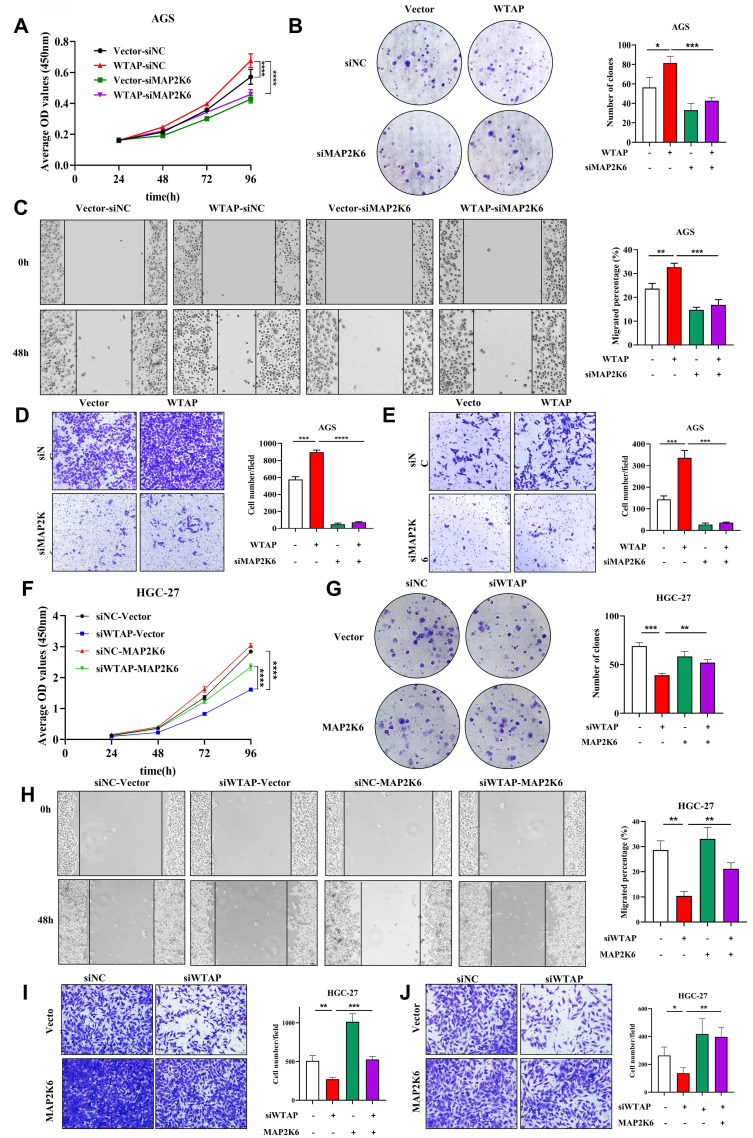
** Oncogenic function of WTAP depends on MAP2K6. (A, B)** CCK-8 and colony formation assay were used to determine the effect of down-regulation of MAP2K6 expression on cell proliferation induced by overexpression of WTAP. **(C, D)** Wound healing assay and Transwell migration assay were used to investigate the effect of down-regulation of MAP2K6 expression on cell migration potency induced by WTAP overexpression. **(E)** Transwell invasion assay was used to clarify the effect of down-regulation of MAP2K6 expression on cell invasion potency induced by overexpression of WTAP. (F-J) The effects of MAP2K6 on WTAP in HGC-27 cells were detected by CCK-8 assay **(F)**, colony formation assay **(G)**, Wound healing assay **(H)**, Transwell migration assay **(I)**, Transwell invasion assay **(J)**.* *P* < 0.05, ** *P* < 0.01, *** *P* < 0.001, **** *P* < 0.0001.

To further elucidate the interaction mechanism between WTAP and MAP2K6, we performed rescue study in GC cell lines. Firstly, we transfected three siRNA sequences targeting different targets of MAP2K6 in AGS cells, and verified the knockdown efficiency of MAP2K6 by qRT-PCR and WB, and found that si-MAP2K6-3 had the most significant inhibition effect (Supplementary Figure 3), so the si-MAP2K6-3 sequence was selected for subsequent experiments. Subsequently, we used qRT-PCR and WB (Supplementary Figure 4) to verify the overexpression efficiency of pcDNA3.1-MAP2K6 in HGC-27 cell line. Next, we transfected pcDNA3.1-vector/pcDNA3.1-WTAP and siRNA-negative control/siRNA-MAP2K6 into AGS cells and siRNA-negative control/siRNA-WTAP and pcDNA3.1-vector/pcDNA3.1-MAP2K6 into HGC-27 cells, respectively or simultaneously. The results of CCK-8 and colony formation assay (Figure 8A, B), Wound healing and Transwell migration assay (Figure 8C, D), and Transwell invasion assay (Figure 8E) respectively suggested that the enhancement of proliferation, migration and invasion ability of AGS cells after overexpression of WTAP could be reduced by down-regulation of MAP2K6 expression level. Conversely, the attenuation of HGC-27 cell proliferation, migration, and invasion caused by WTAP knockdown can be restored by upregulation of MAP2K6 expression (Figure 8F-J). These results suggest that the oncogenic effect of WTAP on GC cells depends on MAP2K6.

## Discussion

Through literature review and database analysis, our research group found that the role of WTAP-mediated m^6^A modification in the pathogenesis of GC has not been completely elucidated. In this study, WTAP expression level in GC tissues were significantly elevated, and there was a significant correlation between high WTAP expression and advanced age, lymph node metastasis, and a high TNM stage. And WTAP could enhance the proliferation, migration and invasion abilities of GC cell lines, respectively. The findings from *in vivo* experiments further demonstrated that suppressing WTAP expression notably impeded the progression of GC growth. Furthermore, the direct downstream target gene with m^6^A modification of WTAP was identified as MAP2K6 by RNA sequencing and MeRIP sequencing. MeRIP-qPCR, luciferase reporter assay, and Co-IF collectively confirmed that WTAP mediates the m^6^A modification of MAP2K6. This study confirmed that both WTAP and MAP2K6 were significantly up-regulated in GC tissues. It was reported for the first time that WTAP promoted the biological functions of GC cell lines by positively regulating MAP2K6 in an m^6^A-dependent manner, and their interaction mechanism was explored in GC cell lines.

As a methylated transferase, several previous studies have shown that WTAP promotes the occurrence and progression of cancer, for example, WTAP expression is significantly up-regulated in esophageal cancer (EC) samples and is remarkably associated with poor prognosis and advanced stage in EC 15; WTAP is highly expressed in hepatocellular carcinoma (HCC), indicating poor prognosis, and can proliferate liver cancer cell proliferation and tumor growth *in vitro* and *in vivo* 16; WTAP is abundantly expressed in colon adenocarcinoma 17; up-regulation of WTAP can induce a malignant phenotype of pancreatic ductal adenocarcinoma 20, etc., which are consistent with our findings that mRNA and protein expression levels of WTAP were increased in GC tissues. Further analysis of clinicopathological correlation of WTAP expression in GC patients showed that the high expression of WTAP may be related to advanced age, poorly differentiation, lymph node metastasis and higher clinical stage, this may prove valuable in the clinical individualized treatment and prognosis judgment. HCC patients with higher levels of WTAP expression were associated with poorer overall survival (OS) and disease-free survival (DFS), and high WTAP expression was an independent prognostic factor for OS and DFS in HCC patients 16. The above-reported result was consistent with our study. These data together indicates that WTAP promotes GC occurrence and development.

In order to verify the effect of WTAP on the biological functions in GC cells, we overexpressed or knocked down WTAP in HGC-27 and AGS cells. Our results confirmed that GC cells were induced to proliferate, migrate and invade when WTAP was overexpressed, while the opposite effect was seen when it was knocked down. And we also found that Inhibition of WTAP expression could suppress the growth of xenograft tumors in nude mice. A study reported that WTAP deficiency inhibited the proliferative capacity of three hepatoma cell lines, overexpression of WTAP resulted in the reversed consequences, in addition, WTAP silencing significantly suppressed the cell migration and invasion 16. Li *et al.* 21 found that WTAP could promote the migration and invasion of pancreatic cancer (PC) cell lines. Similarly, Weng *et al.* 22 expounded that lung cancer cell proliferation, migration and invasion were suppressed, and the cell apoptosis could be expedited when WTAP was inhibited. These results were consistent with ours, which together confirmed that the presence of WTAP may promote characteristics of malignant growth and metastasis, which may contribute to the development of GC.

Then we verified that MAP2K6 was the direct downstream target gene of WTAP with m^6^A modification by high-throughput sequencing and bioinformatics analysis. MAP2K6, located on the 17q24.3 chromosome, is one of the members of MAPK family, and the protein encoded by it contains 334 amino acids with a molecular weight of about 37kD. MAP2K6 is a bispecific protein kinase that transduces cellular and environmental stress signals to its substrate, p38 MAP kinase 23, and plays a vital role in the p38 MAP kinase signaling cascade 24. Studies have shown that the elevated expression level of MAP2K6 could significantly accelerate the cell survival and colony formation ability of nasopharyngeal carcinoma cells 25, and suggested that nasopharyngeal cancer patients had a poor prognosis 26; shRNA-mediated knockdown of MAP2K6 hindered the proliferation of esophageal adenocarcinoma cells and restrains tumor growth 27; The expression level of MAP2K6 was significantly elevated in patients with polycystic ovary syndrome (PCOS) 28. Obviously, these results are consistent with our current research findings on MAP2K6 in GC. The results of MeRIP-qPCR, dual-luciferase reporter gene assay, Co-IF and bioinformatics website suggested that WTAP directly interacted with MAP2K6 in an m^6^A-dependent manner. Through qRT-PCR, WB, Co-IF and rescue study, we successively confirmed that WTAP and MAP2K6 expression were positively correlated, WTAP and MAP2K6 interacted in GC, and WTAP had a positive regulatory effect on MAP2K6. Taken together, it can be concluded from our current research findings that WTAP-mediated m^6^A modification positively regulates MAP2K6 through epigenetic activation, thereby enhancing GC cell proliferation, migration, and invasion.

These findings are sufficient to reveal the significant value of WTAP and MAP2K6 in the pathogenesis and prognosis of GC, provide ideas for the establishment of optimal diagnostic and prognostic biomarkers for GC patients, and have far-reaching guiding significance for the implementation of clinical medicine. More importantly, our study also pointed out the direction for further in-depth exploration of the regulatory role of WTAP-MAP2K6 in GC and other types of cancer, and laid a theoretical foundation for a thorough exploration of the molecular biological process of m^6^A modification.

Admittedly, our current experiment still has some limitations: firstly, the human tissue samples used for clinical validation were all from the same hospital, lacking multi-center clinical data; secondly, this study only carried out a series of experiments in GC cell lines, but not in normal gastric mucosa epithelial cell lines. After further overcoming the above limitations in follow-up studies, we believe that our experimental conclusions will be more convincing and clinically meaningful.

## Conclusions

In conclusion, our findings demonstrate that WTAP-mediated m^6^A modification enhances GC cell proliferation, migration, and invasion by positively regulating MAP2K6, confirming that both WTAP and MAP2K6 are key oncogenes in GC. This is of great significance for the subsequent studies on the pathogenesis of GC and research into new strategies for treating GC clinically.

## Supplementary Material

Supplementary figures and table.

## Figures and Tables

**Table 1:** The expression level of WTAP in GC patients by IHC.

Group	Cases	WTAP expression		Positive Rate (%)	χ^2^	*P*
		Positive	Negative			
Gastric cancer tissues	50	36	14	72	19.360	0.000*
Adjacent normal tissues	50	14	36	28		

* *P*<0.05 represents the p-values with significant differences.

**Table 2:** The relationship between the relative expression level of WTAP and clinicopathological variables in 50 patients with fresh GC tissues.

Clinicopathological data	Cases	WTAP expressions (‾x ± s)	*P*-value
Gender			0.183
Male	41	2.99±1.36	
Female	9	2.47±0.92	
Age (years)			0.014*
≤65	24	2.43±1.18	
>65	26	3.32±1.27	
Tumor size (cm)			0.427
≤4	28	3.02±1.33	
>4	22	2.73±1.27	
Differentiation			0.025^*#^
Poorly	21	3.45±1.27	
Well	24	2.58±1.23	
Lymph node metastasis			0.236
Absent	20	3.15±1.14	
Present	30	2.72±1.39	
TNM Staging			0.028*
I+II	19	2.39±1.15	
III+IV	31	3.20±1.30	
Histology type			0.102
Adenocarcinoma	45	2.98±1.31	
Others	5	2.09±0.93	

* P<0.05 represents the p-values with significant differences, # 5 cases of non-gastric adenocarcinoma (signet ring cell carcinoma/neuroendocrine carcinoma) were excluded.

**Table 3:** The relationship between the relative expression level of WTAP and clinicopathological variables in 50 patients with paraffin GC tissues.

Clinicopathological data	Cases	WTAP expressions		*χ2*	*P*-value
		positive	negative		
Gender				2.635	0.105
Male	41	32	9		
Female	9	4	5		
Age (years)				4.276	0.039*
≤65	24	14	10		
>65	26	22	4		
Tumor size (cm)				0.248	0.594
≤4	28	21	7		
>4	22	15	7		
Differentiation				0.002	0.965^#^
Poorly	21	15	6		
Well	24	17	7		
Lymph node metastasis				4.778	0.029*
Absent	20	11	9		
Present	30	25	5		
TNM Staging				5.702	0.017*
I+II	19	10	9		
III+IV	31	26	5		
Histology type				0.000	1.000
Adenocarcinoma	45	32	13		
Others	5	4	1		

*P<0.05 represents the p-values with significant differences, # 5 cases of non-gastric adenocarcinoma (signet ring cell carcinoma/neuroendocrine carcinoma) were excluded.

## References

[1] Sung H, Ferlay J, Siegel RL (2021). Global Cancer Statistics 2020: GLOBOCAN Estimates of Incidence and Mortality Worldwide for 36 Cancers in 185 Countries. CA: a cancer journal for clinicians.

[2] Zheng RS, Zhang SW, Zeng HM (2022). Cancer incidence and mortality in China, 2016. Journal of the National Cancer Center.

[3] Fukagawa T, Katai H, Mizusawa J (2018). A prospective multi-institutional validity study to evaluate the accuracy of clinical diagnosis of pathological stage III gastric cancer (JCOG1302A). Gastric cancer: official journal of the International Gastric Cancer Association and the Japanese Gastric Cancer Association.

[4] Smyth EC, Verheij M, Allum W, Cunningham D, Cervantes A, Arnold D (2016). Gastric cancer: ESMO Clinical Practice Guidelines for diagnosis, treatment and follow-up. Annals of oncology: official journal of the European Society for Medical Oncology.

[5] Arnold M, Karim-Kos HE, Coebergh JW (2015). Recent trends in incidence of five common cancers in 26 European countries since 1988: Analysis of the European Cancer Observatory. European journal of cancer (Oxford, England: 1990).

[6] Chmiela M, Karwowska Z, Gonciarz W, Allushi B, Stączek P (2017). Host pathogen interactions in Helicobacter pylori related gastric cancer. World journal of gastroenterology.

[7] Yu J, She Y, Yang L (2021). The m(6) A Readers YTHDF1 and YTHDF2 Synergistically Control Cerebellar Parallel Fiber Growth by Regulating Local Translation of the Key Wnt5a Signaling Components in Axons. Adv Sci (Weinh).

[8] Dominissini D, Moshitch-Moshkovitz S, Schwartz S (2012). Topology of the human and mouse m6A RNA methylomes revealed by m6A-seq. Nature.

[9] Liu Z, Zhang J (2018). Human C-to-U Coding RNA Editing Is Largely Nonadaptive. Molecular biology and evolution.

[10] Xie Q, Wu TP, Gimple RC (2018). N(6)-methyladenine DNA Modification in Glioblastoma. Cell.

[11] Jia G, Fu Y, Zhao X (2011). N6-methyladenosine in nuclear RNA is a major substrate of the obesity-associated FTO. Nat Chem Biol.

[12] Yang Y, Hsu PJ, Chen YS, Yang YG (2018). Dynamic transcriptomic m(6)A decoration: writers, erasers, readers and functions in RNA metabolism. Cell Res.

[13] Little NA, Hastie ND, Davies RC (2000). Identification of WTAP, a novel Wilms' tumour 1-associating protein. Human molecular genetics.

[14] Ping XL, Sun BF, Wang L (2014). Mammalian WTAP is a regulatory subunit of the RNA N6-methyladenosine methyltransferase. Cell research.

[15] Zhao H, Xu Y, Xie Y (2021). m6A Regulators Is Differently Expressed and Correlated With Immune Response of Esophageal Cancer. Frontiers in cell and developmental biology.

[16] Chen Y, Peng C, Chen J (2019). WTAP facilitates progression of hepatocellular carcinoma via m6A-HuR-dependent epigenetic silencing of ETS1. Mol Cancer.

[17] Liu X, Liu L, Dong Z (2019). Expression patterns and prognostic value of m(6)A-related genes in colorectal cancer. American journal of translational research.

[18] Tang J, Wang F, Cheng G (2018). Wilms' tumor 1-associating protein promotes renal cell carcinoma proliferation by regulating CDK2 mRNA stability. Journal of experimental & clinical cancer research: CR.

[19] Kuai Y, Gong X, Ding L (2018). Wilms' tumor 1-associating protein plays an aggressive role in diffuse large B-cell lymphoma and forms a complex with BCL6 via Hsp90. Cell communication and signaling: CCS.

[20] Deng J, Zhang J, Ye Y (2021). N(6) -methyladenosine-Mediated Upregulation of WTAPP1 Promotes WTAP Translation and Wnt Signaling to Facilitate Pancreatic Cancer Progression. Cancer research.

[21] Li BQ, Liang ZY, Seery S (2019). WT1 associated protein promotes metastasis and chemo-resistance to gemcitabine by stabilizing Fak mRNA in pancreatic cancer. Cancer letters.

[22] Weng L, Qiu K, Gao W, Shi C, Shu F (2020). LncRNA PCGEM1 accelerates non-small cell lung cancer progression via sponging miR-433-3p to upregulate WTAP. BMC pulmonary medicine.

[23] Rasmussen MH, Lyskjær I, Jersie-Christensen RR (2016). miR-625-3p regulates oxaliplatin resistance by targeting MAP2K6-p38 signalling in human colorectal adenocarcinoma cells. Nature communications.

[24] Matsumoto T, Kinoshita T, Matsuzaka H (2012). Crystal structure of non-phosphorylated MAP2K6 in a putative auto-inhibition state. Journal of biochemistry.

[25] Li Z, Fu J, Li N, Shen L (2018). Quantitative proteome analysis identifies MAP2K6 as potential regulator of LIFR-induced radioresistance in nasopharyngeal carcinoma cells. Biochemical and biophysical research communications.

[26] Li Z, Li N, Shen L (2018). MAP2K6 is associated with radiation resistance and adverse prognosis for locally advanced nasopharyngeal carcinoma patients. Cancer management and research.

[27] Lin S, Liu K, Zhang Y (2018). Pharmacological targeting of p38 MAP-Kinase 6 (MAP2K6) inhibits the growth of esophageal adenocarcinoma. Cellular signalling.

[28] Wu G, Xia J, Yang Z (2022). CircASPH promotes KGN cells proliferation through miR-375/MAP2K6 axis in Polycystic Ovary Syndrome. Journal of cellular and molecular medicine.

