# Supplementary material for: WTAP-Mediated N6-Methyladenosine Modification Promotes Gastric Cancer Progression by Regulating MAP2K6 Expression
Source: J Cancer. 2025 Jan 27;16(5):1420–37. doi: 10.7150/jca.98559 (PMC11843227; doi:10.7150/jca.98559)
Supplement: Supplementary file 1 — Supplementary figures and table. [file jcav16p1420s1.pdf]

Supplementary Figures

Supplementary Figure 1: Basic expression of WTAP protein in wild-type GES-1, HGC-27, AGS, MKN7 and MKN74 cells.

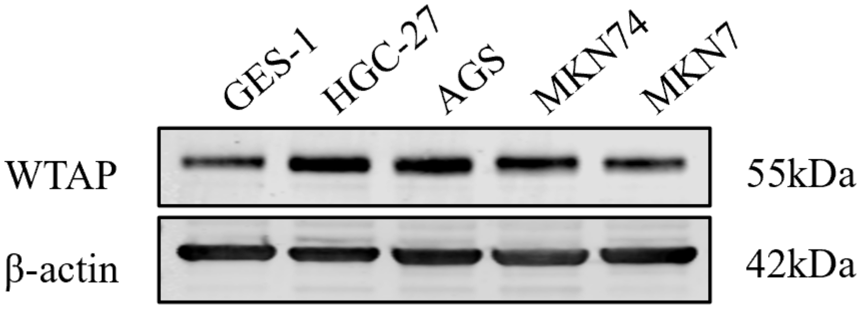

Supplementary Figure 2: The mRNA expression levels of CNTN1, LHX9, SYT1, MAP2K6 and PRUNE2 in GC and adjacent normal tissues in TCGA and GEPIA2 websites.

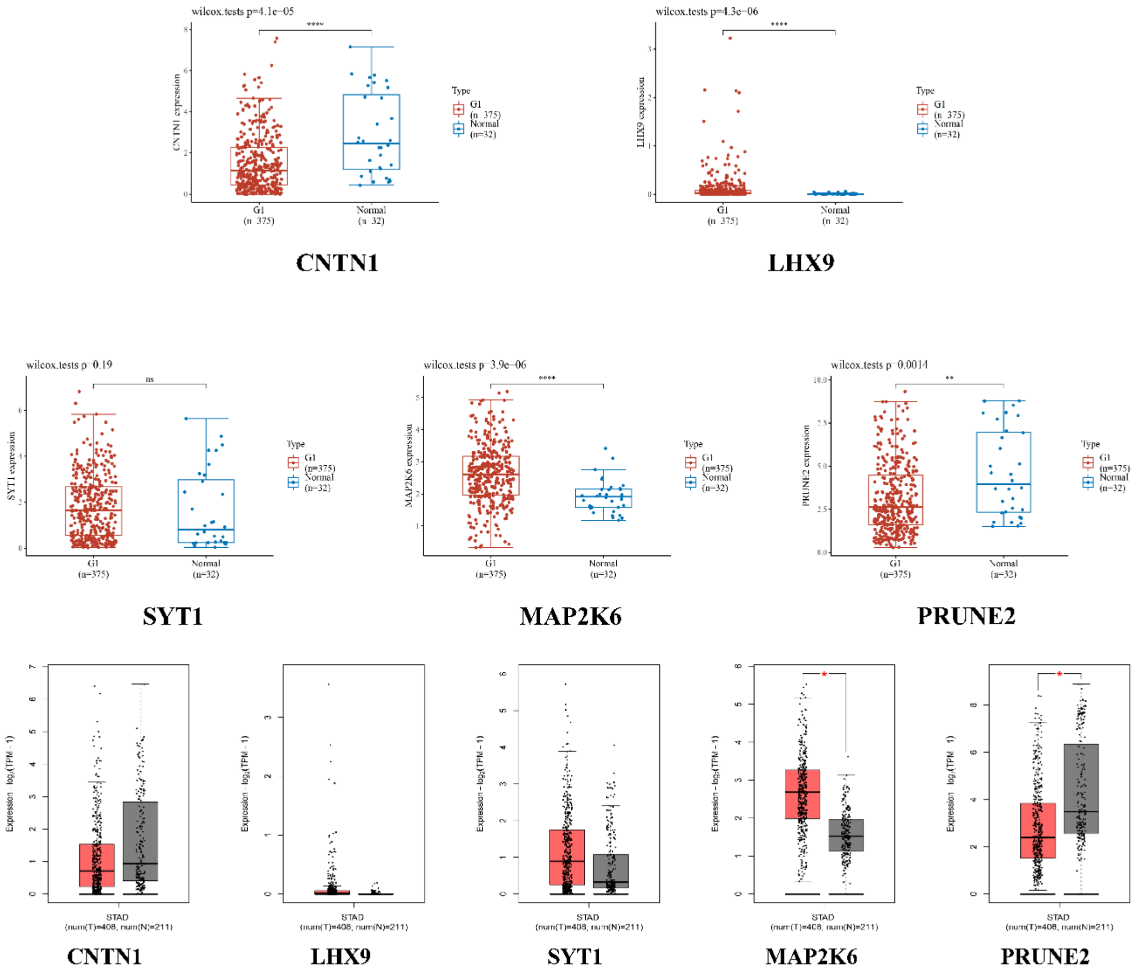

Supplementary Figure 3: Detection of knockdown efficiency of MAP2K6 in AGS cells.

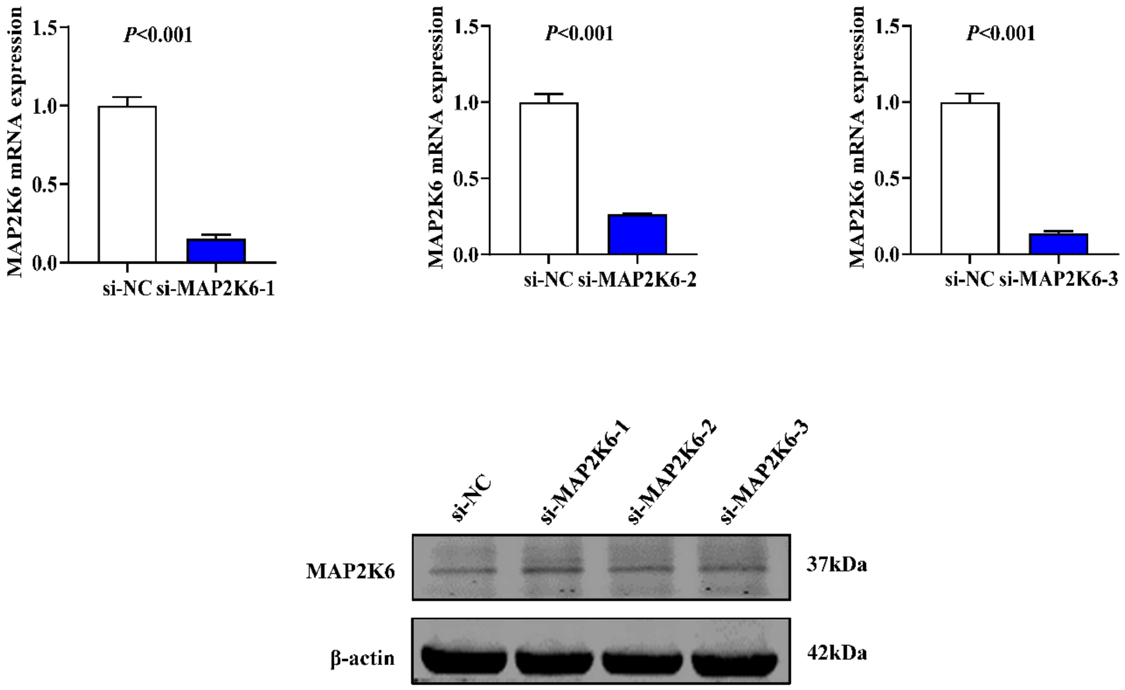

Supplementary Figure 4: Detection of overexpression efficiency of MAP2K6 in HGC-27 cells.

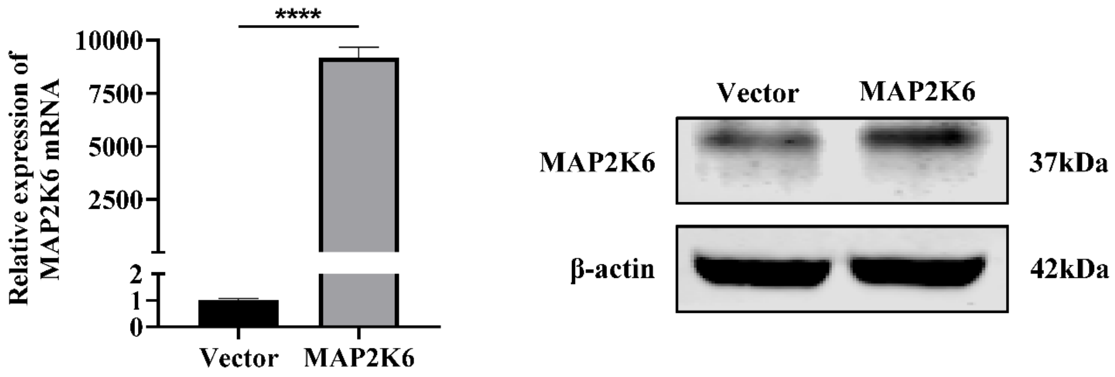

Supplementary Table 1 The primer sequences of WTAP, β-actin, CNTN1, LHX9, SYT1, MAP2K6, PRUNE2

**Gene name**	**primer sequences**	
WTAP	Forward	5′-ACTGGCCTAAGAGAGTCTGAAG-3′
	Reverse	5′-GTTGCTAGTCGCATTACAAGGA-3′
β-actin	Forward	5′-CTCCATCCTGGCCTCGCTGT-3′
	Reverse	5′-GCTGTCACCTTCACCGTTCC-3′
CNTN1	Forward	5′-GGATGGTCAGAAGCACTGAAG-3′
	Reverse	5′-CAAATCGCCGTTTATCCATTGTG-3′
LHX9	Forward	5′-GCTGTGGACAAACAGTGGC-3′
	Reverse	5′-CTCAGGTGGTAGACAGAGTCTC-3′
SYT1	Forward	5′-GTGAGCGAGAGTCACCATGAG-3′
	Reverse	5′-CCCACGGTGGCAATGGAAT-3′
MAP2K6	Forward	5′-GAAGCATTTGAACAACCTCAGAC-3′
	Reverse	5′-CCTGGCTATTTACTGTGGCTC-3′
PRUNE2	Forward	5′-CAACGCGCCAAATCTAAACTG-3′
	Reverse	GTTCAGCACTGGTAAACACAGA-3′
